# Supplementary material for: Endogenous LRRK2 and PINK1 function in a convergent neuroprotective ciliogenesis pathway in the brain
Source: Proc Natl Acad Sci U S A. 2025 Jan 28;122(5):e2412029122. doi: 10.1073/pnas.2412029122 (PMC11804522; doi:10.1073/pnas.2412029122)
Supplement: Supplementary file 1 — Appendix 01 (PDF) [file pnas.2412029122.sapp1.pdf]

The Arrive Essential 10

These items are the basic minimum to include in a manuscript. Without this information, readers and reviewers cannot assess the reliability of the findings.

1. **Study Design**
   For each experiment, provide brief details of study design including:
   a. The groups being compared, including control groups. If no control group has been used, the rationale should be stated.
   b. The experimental unit (e.g. a single animal, litter, or cage of animals)
2. **Sample size**
   a. Specify the exact number of experimental units allocated to each group, and the total number in each experiment. Also indicate the total number of animals used.
   b. Explain how the sample size was decided. Provide details of any a priori sample size calculation, if done.

All experiments were conducted according to the ASPA and ASRU guidelines (The study performed were previously approved by the University of Dundee Ethical Review Committee and carried out under a U.K. Home Office project licence, both in Dundee and Cardiff.

Study 1. Behavioural analysis of PINK1^{WT}/LRRK2^{WT}; PINK1^{WT}/LRRK2^{RC}; PINK1^{KO}/LRRK2^{WT} and PINK1^{KO}/LRRK2^{RC}

Study plan YL05
Battery of motor and behavioural test to assess possible phenotype of the 4 different genotypes. Followed by immunofluorescence analysis of microglia and dopaminergic neurons in the brain.
Control group is PINK1^{WT}/LRRK2^{WT} and all groups were compared between each other (ordinary 2-way ANOVA, Sidak's multiple comparison test).
Experimental unit: single animal
N Numbers (female, males):

- PINK1^{WT}/LRRK2^{WT}: 15 (4,11)
- PINK1^{WT}/LRRK2^{RC}: 16 (6,10)
- PINK1^{KO}/LRRK2^{WT}: 15 (4,11)
- PINK1^{WT}/LRRK2^{RC}: 15 (7,8)

N numbers calculated a priori according to previous work and published literature.
This study was performed at the School of bioscience, Cardiff University

Study 2. Biochemical analysis of PINK1^{WT}/LRRK2^{WT}; PINK1^{WT}/LRRK2^{RC}; PINK1^{KO}/LRRK2^{WT} and PINK1^{KO}/LRRK2^{RC}

Study plan SB02
Biochemical analysis by Western Blot and ELISA (pSer65 Ub) to assess a possible interplay between the PINK1 and LRRK2 signalling pathways in the 4 mouse lines.
Control group is PINK1^{WT}/LRRK2^{WT} and all groups were compared between each other (ordinary 2-way ANOVA, Sidak's multiple comparison test, MLi2 treated group not used for statistical test).
Experimental unit: single animal
N Numbers (vehicle, MLi2):

- PINK1^{WT}/LRRK2^{WT}: 5 (4,1)
- PINK1^{WT}/LRRK2^{RC}: 6 (4,2)

- $PINK1^{KO}/LRRK2^{WT}$: 6 (4,2)
- $PINK1^{WT}/LRRK2^{RC}$: 6 (4,2)

N numbers calculated a priori according to previous work and published literature.
This study was erformed in Dundee.

Study 3. Ciliogenesis analysis of $PINK1^{WT}/LRRK2^{WT}$; $PINK1^{WT}/LRRK2^{RC}$; $PINK1^{KO}/LRRK2^{WT}$ and $PINK1^{KO}/LRRK2^{RC}$

Study plan EB04
Analysis of ciliary and signalling defect by Immunofluorescence and Fluorescence in situ hybridization (FISH).
Control group is $PINK1^{WT}/LRRK2^{WT}$ and all groups were compared between each other (ordinary 2-way ANOVA, Tukey's multiple comparison test, MLi2 treated group not used for statistical test).
Experimental unit: single animal
N Numbers

- $PINK1^{WT}/LRRK2^{WT}$: 4
- $PINK1^{WT}/LRRK2^{RC}$: 4
- $PINK1^{KO}/LRRK2^{WT}$: 3
- $PINK1^{WT}/LRRK2^{RC}$: 3

N numbers calculated a priori according to previous work and published literature. This study was performed in Dundee (animal husbandry, culling and processing) and Stanford (immunohistochemistry and analysis).

3. Inclusion and exclusion criteria

a. Describe any criteria used for including and excluding animals (or experimental units) during the experiment, and data points during the analysis. Specify if these criteria were established a priori. If no criteria were set, state this explicitly.
b. For each experimental group, report any animals, experimental units or data points not included in the analysis and explain why. If there were no exclusions, state so.
c. For each analysis, report the exact value of n in each experimental group.

In study 2, one mouse ($PINK1^{WT}/LRRK2^{RC}$) was culled on welfare grounds and replaced by an animal of the same genotype.

This mouse (ID 343348) was culled due to an abscess/infected wound. Mild severity.
Other two mice (not experimental, but cage-mates and spare) were culled.
Mouse 336627 was culled due to a decrease of more than 10% of its body weight and a body condition score (BCS) of 2.5. Post-mortem analysis didn't reveal anything. General deterioration due to age. Mild severity.
Mouse 336629 was culled due to a worsening of general health (swollen eye, holding her ears flat, hunching in at the rear), BCS = 2.5. Post-mortem analysis didn't reveal anything. General deterioration due to age. Mild severity

These events were not that unexpected, as this was an ageing study and we knew that a deterioration of general health condition could occur. Which is the reason why we decided to include 1 spare mouse per genotype.

No other mouse was excluded from the analysis.

4. Randomisation

a. State whether randomisation was used to allocate experimental units to control and treatment groups. If done, provide the method used to generate the randomisation sequence.
b. Describe the strategy used to minimise potential confounders such as the order of treatments and measurements, or animal/cage location. If confounders were not controlled, state this explicitly.

Randomisation was used to assign a treatment (MLi2 or vehicle) to each mouse in study 2. Random numbers (1 to 23) were generated in excel and assigned to each mouse. For each genotype, the two mice with the highest number were injected with MLi2 (for the $PINK1^{WT}/LRRK2^{WT}$ control line only one animal was assigned to Mli2 group).

Cofounders were not controlled.

5. Blinding

Describe who was aware of the group allocation at the different stages of the experiment (during the allocation, the conduct of the experiment, the outcome assessment, and the data analysis)

Blinding was performed for Study 2 and study 3.
For study 2, the person injecting the mice with either vehicle or MLi2 was blinded regarding the genotype of the mice.

For study 3, cilia analysis performed by collaborators in Stanford was done blind and the genotype of each mouse revealed at the end of the quantification. The person preparing the mice for fixation and shipment was not blinded.

6. Outcome measures

a. Clearly define all outcome measures assessed (e.g. cell death, molecular markers, or behavioural changes).
b. For hypothesis-testing studies, specify the primary outcome measure, i.e. the outcome measure that was used to determine the sample size.

Study 1. **Behavioural and motor analysis**
Weight (grams)
Righting time in negative geotaxis assay (seconds)
Grip strength (AU)
Gait analysis: Stride length (cm), forelimb and hindlimb base width (cm) and overlap (cm)
Balance beam: Forelimb, hindlimb and total foot slips (number), turn time (seconds)
Rotarod: time to fall
Colourimetric immunohistochemistry
Microglia: Iba1 positive cells per field of view

Striatum: Medium spiny neuron (DARPP32) staining (AU) and total striatal volume (mm^3)

Study 2. **Biochemical analysis**
Assessment of various PINk1 and LRRk2 components by WB
Measure of pSer65 Ub by ELISA

Study 3. **Ciliogenesis analysis**
Cilia: Number of cilia/ cell by IF
GDNF: number of GDNF dots/cell by FISH

7. Statistical methods

a. Provide details of the statistical methods used for each analysis, including software used.
b. Describe any methods used to assess whether the data met the assumptions of the statistical approach, and what was done if the assumptions were not met.

All statistical analysis was performed in Prism 10.

Study 1. **Behavioural analysis of $PINK1^{WT}/LRRK2^{WT}$; $PINK1^{WT}/LRRK2^{RC}$; $PINK1^{KO}/LRRK2^{WT}$ and $PINK1^{KO}/LRRK2^{RC}$**
Control group is $PINK1^{WT}/LRRK2^{WT}$ and all groups were compared using ordinary 2-way ANOVA, Sidak's multiple comparison test.

Study 2. **Biochemical analysis of $PINK1^{WT}/LRRK2^{WT}$; $PINK1^{WT}/LRRK2^{RC}$; $PINK1^{KO}/LRRK2^{WT}$ and $PINK1^{KO}/LRRK2^{RC}$**
Control group is $PINK1^{WT}/LRRK2^{WT}$ and all groups were compared using ordinary 2-way ANOVA, Sidak's multiple comparison test.
The comparison done are:

- $PINK1^{WT}/LRRK2^{WT}$ vs $PINK1^{KO}/LRRK2^{WT}$
- $PINK1^{WT}/LRRK2^{WT}$ vs $PINK1^{WT}/LRRK2^{RC}$
- $PINK1^{KO}/LRRK2^{WT}$ vs $PINK1^{KO}/LRRK2^{RC}$
- $PINK1^{WT}/LRRK2^{RC}$ vs $PINK1^{KO}/LRRK2^{RC}$

MLi2 group was not included in the comparison but used only as positive control for LRRK2 substrate phosphorylation

Study 3. **Ciliogenesis analysis of $PINK1^{WT}/LRRK2^{WT}$; $PINK1^{WT}/LRRK2^{RC}$; $PINK1^{KO}/LRRK2^{WT}$ and $PINK1^{KO}/LRRK2^{RC}$**
Control group is $PINK1^{WT}/LRRK2^{WT}$ and all groups were compared between each otherOrdinary 2-way ANOVA, Tukey's multiple comparison test, comparing cell mean with every other cell mean.

8. Experimental animals

a. Provide species-appropriate details of the animals used, including species, strain and substrain, sex, age or developmental stage, and, if relevant, weight.
b. Provide further relevant information on the provenance of animals, health/immune status, genetic modification status, genotype, and any previous procedures.

The mice used derive from the C57 line, substrain J.
The four genotypes used are:

- $PINK1^{WT}/LRRK2^{WT}$
- $PINK1^{WT}/LRRK2^{RC}$
- $PINK1^{KO}/LRRK2^{WT}$
- $PINK1^{WT}/LRRK2^{RC}$

And were obtained by crossing the appropriate mutant mice.
For Study 1 and Study 2 mice were used at 10.5 months old. Study 3 was performed on 5 months old mice.
More details on the mice can be found in the KRT in the supplementary info of the paper and each mouse line has been deposited on MGI.

Animals were genotyped according to the following protocol:

LRRK2 R1441C
LRRK2 R1441C 9403: CTGCAGGCTACTAGATGGTCAAGGT
LRRK2 R1441C 9404: CTAGATAGGACCGAGTGTCGCAGAG
Results:
WT: 386bp
MUT: 520bp

PINK1 KO
EB0088-26: CTGCCCTCAGGGTCTCTAATGC
EB0088-27: GGAAGGAGGCCATGGAAATTGT
Neo3a: GCAGCGCATCGCCTTCTATC
Results:
WT: 296bp
Mut: 193bp

Alternatively for Transnetyx Genotyping use the following probes:
LRRK2 R1441C - Lrrk2-22 Mut
PINK1 KO - Pink1-1 WT and PINK1-1 KO

More details on the mice are attached below:
Study 1 – YL05

Mouse #	Dundee #	Sex	Lrrk2	Pink1	Weight
97	40	M	Wt	Hom	22.5
59	148	M	Hom	Hom	34.6
60	149	M	Hom	Hom	31.6
61	150	M	Hom	Hom	32.6
62	151	F	Hom	Hom	24.5
63	152	F	Hom	Hom	23.1
70	153	M	Hom	Wt	29.3
71	154	M	Hom	Wt	25.5
72	155	M	Hom	Wt	25.8
73	156	F	Hom	Wt	25.4
74	157	F	Hom	Wt	22.4
75	158	M	Hom	Wt	27.7
76	159	M	Hom	Wt	29.3
77	160	M	Hom	Wt	25.6
78	161	M	Hom	Wt	25.1
79	162	F	Hom	Wt	21.9
80	163	F	Hom	Wt	24.2
81	164	F	Hom	Wt	21.1
82	165	F	Hom	Wt	20.3
83	336	M	Hom	Wt	30.3
84	337	M	Hom	Wt	28.8
85	338	M	Hom	Wt	29.4
98	345	M	Wt	Hom	25.3
99	346	M	Wt	Hom	25.9
100	347	M	Wt	Hom	23
44	409	M	Wt	Wt	29.4
45	410	M	Wt	Wt	29.5
46	411	M	Wt	Wt	30.1
47	412	M	Wt	Wt	29.4
48	413	M	Wt	Wt	28.1
49	414	M	Wt	Wt	27
50	415	M	Wt	Wt	27.8
51	416	M	Wt	Wt	27.7
86	418	M	Wt	Hom	25.2
87	419	M	Wt	Hom	28.9
88	420	M	Wt	Hom	27.5
89	421	F	Wt	Hom	21.7
90	422	F	Wt	Hom	22.4
91	423	F	Wt	Hom	23.3
92	424	F	Wt	Hom	21.4
53	427	M	Wt	Wt	29.2
54	428	M	Wt	Wt	27.8
52	436	M	Wt	Wt	32
93	437	M	Wt	Hom	27.6
94	438	M	Wt	Hom	27.9
95	439	M	Wt	Hom	22.9
96	440	M	Wt	Hom	23.2
64	441	M	Hom	Hom	32.3
65	442	M	Hom	Hom	29.9
66	443	M	Hom	Hom	29.8
67	447	F	Hom	Hom	20.8
68	448	F	Hom	Hom	21.4
69	449	F	Hom	Hom	21.1
40	907	F	Wt	Wt	25.7
41	908	F	Wt	Wt	23.8
42	909	F	Wt	Wt	23.7
43	910	F	Wt	Wt	22.2
55	911	M	Hom	Hom	28.8
56	912	M	Hom	Hom	29.3
57	913	F	Hom	Hom	22.1
58	914	F	Hom	Hom	23.3

Study 2 – SB02

Genotype	Global 1	assigned numebr	exp. ID	Age	Sex	Weight at cullling	notes
r1441c-wt	pink1-wt	3	1	345	Male	30.8	
r1441c-wt	pink1-wt	5	2	345	Male	31.4	
r1441c-wt	pink1-hom	6	3	292	Male	31.2	
r1441c-wt	pink1-hom	12	4	292	Female	26.6	
r1441c-hom	pink1-wt	24	5	294	Male	35.4	
r1441c-hom	pink1-wt	26	6	292	Male	32	
r1441c-hom	pink1-hom	13	7	411	Female	25.2	
r1441c-hom	pink1-hom	16	8	411	Female	32.9	
r1441c-wt	pink1-wt	1	9	345	Female	22.3	
r1441c-wt	pink1-hom	8	10	292	Female	24.1	
r1441c-hom	pink1-wt	25	11	292	Female	25.1	
r1441c-hom	pink1-hom	19	12	411	Male	38.4	
r1441c-wt	pink1-hom	7	13	292	Male	32.3	
r1441c-hom	pink1-wt	23	14	294	Male	30.5	
r1441c-hom	pink1-hom	15	15	411	Female	25.5	
r1441c-wt	pink1-wt	2	16	345	Female	24.2	
r1441c-wt	pink1-hom	10	17	292	Male	33.6	
r1441c-hom	pink1-wt	20	18	294	Male	35	
r1441c-hom	pink1-hom	18	19	411	Male	34.6	
r1441c-wt	pink1-wt	4	20	345	Male	38.1	
r1441c-wt	pink1-hom	11	21	292	Male		culled on welfare ground
r1441c-hom	pink1-wt	21	22	294	Male	33.3	
r1441c-hom	pink1-hom	17	23	411	Female	27.4	
r1441c-wt	pink1-hom	9	24	292	Male	30	replacement for 343348

Study 3 -EB04

LRRK2	PINK1	Age	Sex	weight
hom	pink1-wt	147	Female	23
hom	pink1-wt	145	Female	22
hom	pink1-wt	145	Male	30
wt	pink1-wt	143	Male	32
wt	pink1-wt	143	Male	34.5
wt	pink1-wt	143	Male	36.6
wt	pink1-wt	151	Male	37.4
hom	pink1-wt	143	Male	34.7
hom	Pink1-hom	153	Male	30.9
hom	Pink1-hom	153	Male	31.7
hom	Pink1-hom	153	Male	30.7
wt	Pink1-hom	153	Male	30.16
wt	Pink1-hom	153	Male	29.3
wt	Pink1-hom	153	Male	30.9

9. Experimental procedures

For each experimental group, including controls, describe the procedures in enough detail to allow others to replicate them, including:

a. What was done, how it was done and what was used.
b. When and how often.
c. Where (including detail of any acclimatisation periods).
d. Why (provide rationale for procedures).

Below detailed methods. More information can be found in the link for the relative protocols.

Mice behavioural and motor test

Behavioural tests were conducted on 10.5 months old mice. Mice were weighed before the start of behavioural tests to make a comparison between genotypes.

Negative geotaxis was assessed by placing the animal onto a mesh grid (30 x 30 cm). The time taken to rotate through 180° from a head down position was recorded as a measure of proprioception.

Grip strength was measured using a grip meter modified from GSM1054 model (Linton Instrumentation) as previously described [1]. In two consecutive trials, the mouse was held by the tail while body supported and lowered onto the instrument until it gripped the two bars. The mouse was pulled by the base of the tail until the grip loosened. The applied force at which the mouse released the bars was recorded and averaged across the two trials.

Gait analysis, rotarod and balance beam were conducted as described in [47. Briefly, gait analysis was carried out using the footprint test. The animal was placed in a clear Perspex corridor apparatus (65 cm L x 15 cm W) and trained to run towards a dark goal box at the end of the corridor until it could reach the box without encouragement. For testing, a paper strip was placed in the corridor and, to leave footprints, the mouse's paws were painted with non-toxic, water-based paints in two different colours to identify the front paws versus the hind paws. The mouse was allowed to run the entire length of the apparatus and reach the goal box. The stride length, the stride width and the overlap were measured using 4 paws print, allowing to average 3 values for each measurement.

Rotarod was carried out using a commercial Rotarod apparatus (Ugo Basile, model 47600). After 5 sessions of training conducted over 5 consecutive days (max 5 min/session), mice were tested in two different trials (accelerating rod from 5 rpm to 44 rpm in each trial). The latency to fall was recorded and averaged across the two trials. For the balance beam, mice were trained on an elevated bridge (1 m in length, 17% angle of ascent, with 1.5 to 0.5 cm tapers across the width) with a dark house box at the high end. During the first day of training, the mouse was placed in front of the house box and allowed to enter the box. The distance from the house box was progressively increased until the low end of the beam. The mouse was then placed at the low end of the beam, facing away from the house box, and encouraged to turn around and transverse the beam until the house box. The test was carried out in two consecutive trials, conducted 1 hour apart, and videotaped to allow analysis. The mouse was placed at the low end of the beam, facing away the house box. The time taken to turn around, transverse the beam and the number of foot slips were recorded and averaged across the two trials.

MLi-2 treatment in mice

To ensure LRRK2-dependent phosphorylation of Rabs, mice were treated with the LRRK2 inhibitor MLi-2. The compound was administered to mice via subcutaneous injection as described (dx.doi.org/10.17504/protocols.io.bezdjf26). MLi-2 was resuspended in a 40% Hydroxypropyl-β-Cyclodextran (Average Mw ~1,460) solution at 6 mg/ml. It was then administered by subcutaneous injection at 30 mg/kg. The Dundee-synthesised MLi-2 (MTA-free) was used for this experiment. Mice were culled by cervical dislocation 2 hours after the injections, tissue collected and lysed as outlined above.

Cell and tissue lysis and immunoblotting

Cells were quickly washed on ice in PBS, then lysed in buffer containing Tris-HCl (50 mm, pH 7.5), EDTA (1 mM), EGTA (1 mM), Triton (1% w/v), sodium orthovanadate (1 mM), sodium glycerophosphate (10 mM), sodium fluoride (50 mM), sodium pyrophosphate (10 mM), sucrose (0.25 mM), protease inhibitor cocktail (Roche), phoSTOP (Roche), and chloroacetamide (200 mM). Tissues were instead collected and snap frozen in liquid nitrogen. They were then weighted, quickly thawed on ice in a 10-fold volume excess of ice cold lysis buffer. Tissues were homogenised using a POLYTRON homogenizer (KINEMATICA), employing three rounds of 10s homogenization with 10s

intervals on ice. Lysates either from cells or tissues, were incubated for 30 min on ice. Samples were spun at 17000 g in an Eppendorf 5417R centrifuge for 30 min at 4°C. Supernatants were collected, and protein concentration was determined by using the Bradford kit (Pierce).
Protein lysates were subjected to SDS–PAGE (4–12% Bis-Tris gel or 12% Tris glycine) and transferred onto nitrocellulose membranes. Membranes were then blocked for 1 h in Tris-buffered saline with 0.1% Tween (TBST) containing 5% (w/v) milk and subsequently probed with the indicated antibodies in TBST containing 5% (w/v) BSA overnight at 4°C. Detection was performed using appropriate secondary antibodies (1:10000) and scanned using Li-COR Odyssey CLx imaging system. More details can be found on protocols.io (dx.doi.org/10.17504/protocols.io.ewov14znkvr2/v2). Signal intensity was quantified using the Image Studio Software and normalised versus the unphosphorylated protein or the loading control. The amount of protein loaded in each lane is reported for each blot.

pSer65 Ub ELISA
Phosphorylation of Ub at Ser65 by PINK1 was monitored *in vivo* by enzyme linked immunosorbent assay (ELISA) as previously described by Watzlawik and colleagues [54]. MSD plates were coated overnight with 30 µl/well of 200 mM sodium carbonate buffer (pH 9.7) containing 1 µg/ml of rabbit monoclonal pSer65-Ub antibody. The next morning plates were washed twice with ELISA washing buffer (150 mM Tris, pH 7.4, 150 mM NaCl, 0.1% Tween-20) by plate inversion and gentle tapping on paper towels (not by pipette aspiration). Plates were then blocked with ELISA blocking buffer (150 mM Tris, pH 7.4, 150 mM NaCl,0.1% Tween-20, 1% BSA) for 1h at room temperature. All samples were run in duplicates and diluted in blocking buffer. 30 µg of total protein were loaded per well for all mouse tissues in a total volume of 30 µl per well. Detergent volumes were adjusted across all samples. Antigens were incubated for 2 h at room temperature on a microplate mixer at 600 rpm and three washing steps were then performed as described before. Mouse total Ub antibody (clone P4D1; Thermo Fisher #14-6078-37) was subsequently added as detecting antibody at a final concentration of 5 µg/ml in blocking buffer in 30 µl total volume per well. After three washing steps, 50 µl/well of 1 µg/ml of SULFO-TAG labelled goat anti-mouse antibody (MSD, R32AC-1) in blocking buffer were added and incubated for 1 h at room temperature on a microplate mixer at 500 rpm. After another three washing steps, 150 µl MSD GOLD Read Buffer (MSD, R92TG-2) were finally added to each well and the plate read on a MESO QuickPlex SQ 120 reader.

Fluorescence in situ hybridization (FISH)
RNAscope fluorescence in situ hybridization was conducted as described herein: (bio-protocol.org/prep1423) [51, 55]. The RNAscope Multiplex Fluorescent Detection Kit v2 (#323100, Advanced Cell Diagnostics) was used as per the manufacturer with RNAscope 3-plex Negative Control Probe (#320871) or probe Mm-Gdnf-C1 (#421951). The Mm-Gdnf-C1 probe was diluted 20 X in a buffer containing 6x saline-sodium citrate (SSC), 0.2% lithium dodecyl sulfate, and 20% Calbiochem OmniPur Formamide. Fluorescent visualization of the hybridized probes was achieved using Opal 690 (Akoya Biosciences). Brain slices were blocked with 1% BSA and 2% FBS in Tris-buffered saline with 0.1% Triton X-100 for 30 min. They were then incubated overnight at 4°C with primary antibodies in TBS containing 1% BSA and 1% DMSO. This was followed by treatment with secondary antibodies, diluted in TBS with 1% BSA and 1% DMSO, including 0.1 µg/ml DAPI (Sigma) for 2h at room temperature. Finally, sections were mounted using Fluoromount G and glass coverslips.

Mouse brain immunohistochemistry - colorimetric analysis
For Iba1 and DARPP-32 staining, mice were terminally anesthetised with sodium pentobarbital (Dolethal, I.P.) and perfused with PBS and PFA 4%. Brains were fixed overnight in 4% PFA at 4°C, washed and left in 30% sucrose for 24h at 4°C. Brains were sliced into 35 µm-thick slices using a freezing microtome and stored at -20°C until processing for immunohistochemistry. Free-floating sections were rinsed (3X) with TBS for 10 min and incubated with quenching solution (3% H2O2,

10% Methanol in TBS) for 15 min. Sections were subsequently rinsed (3X) in TBS for 10 min and incubated with blocking solution (5% normal goat serum, TBS-Triton 0.1%) for 1 h at room temperature. Incubation with primary antibodies was performed overnight at 4°C. The following day, sections were rinsed (3X) with TBS Triton 0.1% for 10 min and incubated with the secondary antibodies for 2 h at room temperature. Sections were subsequently rinsed (3X) in TBS-Triton 0.1% for 10 min and incubated for 2 h at room temperature with avidin-peroxidase complex (ABC kit, PK4000, Vector). 3,3'-diaminobenzidine (DAB, Sigma) was applied to the slices to visualise Iba1 and DARPP-32 positive cells. Imaged were obtained using a bright-field microscope (Macro/Micro Imaging System, Leica) under a 40X objective and analysed using Fiji.

Mouse brain immunohistochemistry – _fluorescence analysis

Analysis of primary cilia in the mouse brain striatum was performed as previously described (dx.doi.org/10.17504/protocols.io.bnwimfce). Mice were terminally anaesthetised using a commercial solution of sodium pentobarbital (Dolethal, I.P.), before being perfused with PBS and 4% PFA. The brain was then dissected, fixed overnight in 4% PFA at 4°C, washed and left in 30% sucrose for 48h at 4°C. Whole brains were subsequently embedded in 22 x 22 x 20 mm moulds containing O.C.T. compound and kept at -80°C until sectioning. Sections of the mouse striatum were then obtained with a cryostat with a cutting thickness of 16 µm. Frozen sections were thawed at RT for 15 min and gently washed (2X) with PBS for 5 min. For antigen retrieval, slides were incubated with 10 mM sodium citrate buffer pH 6.0 (preheated to 95° C) for 15 minutes at 95° C. Sections were permeabilized with 0.1% Triton X-100 in 1X PBS at RT for 15 min. Sections were blocked with 2% FBS and 1% BSA in PBS for 2 hr at RT and were then incubated overnight at 4°C with primary antibodies. The following day, sections were incubated with secondary antibodies at RT for 2 hr. Donkey highly cross-absorbed H + L secondary antibodies conjugated to Alexa 488 and Alexa 568 were used at a 1:2000 dilution. Nuclei were stained with 0.1 µg/ml DAPI (Sigma). Stained tissues were overlaid with Fluoromount G and a glass coverslip. All antibody dilutions for tissue staining included 1% DMSO to help antibody penetration. Images were obtained using a Zeiss LSM 900 confocal microscope with a 63 x 1.4 oil immersion objective. Image visualisations and analyses were performed using Fiji.

1. Dunnett, S.B., E.M. Torres, and L.E. Annett, *A lateralised grip strength test to evaluate unilateral nigrostriatal lesions in rats.* Neurosci Lett, 1998. **246**(1): p. 1-4.
2. McWilliams, T.G., et al., *Basal Mitophagy Occurs Independently of PINK1 in Mouse Tissues of High Metabolic Demand.* Cell Metab, 2018. **27**(2): p. 439-449 e5.

10. Results

For each experiment conducted, including independent replications, report:
a. Summary/descriptive statistics for each experimental group, with a measure of variability where applicable (e.g. mean and SD, or median and range).
b. If applicable, the effect size with a confidence interval.

All data, curated and raw, are available in Zenodo, an open access depository, at the following link: https://zenodo.org/communities/asaphub/records?q=&l=list&p=1&s=10&sort=newest

11. Ethical statement

Provide the name of the ethical review committee or equivalent that has approved the use of animals in this study, and any relevant licence or protocol numbers (if applicable). If ethical approval was not sought or granted, provide a justification.

Need to add PPL number and protocols used for these studies, PPL approved internally by UoD AWERB (assume also Cardiff) and by ASRU……

Welfare and ethical use of animals committee remit and terms of reference can be found at:

https://www.dundee.ac.uk/corporate-information/welfare-and-ethical-use-animals-committee

Ethical statements of the Cardiff university can be found at:

https://www.cardiff.ac.uk/research/our-research-environment/integrity-and-ethics/animal-research

The statement on the use of animals in research at the University of Dundee can be found at:

https://www.dundee.ac.uk/corporate-information/statement-use-animals-research

The Dundee University culture of care in animal research can be found at:

https://www.dundee.ac.uk/corporate-information/our-culture-care-animal-research

12. Housing and husbandry

Provide details of housing and husbandry conditions, including any environmental enrichment

Information to report	Breeding Unit	Experimental unit
**Cage/tank/housing system (type and dimensions)**	Thoren IVC small cage filter top 19.56 x 30.91 x 13.34cm<br>Thoren IVC large cage filter top 30.80 x 30.80 x 14.29cm	Tecniplast IVC large Greenline 34 W x 34 L x 18 D, Tecniplast IVC small Greenline 18 W x 38 L x 18 D, Tecniplast IVC Blue line 542 cm2 x 84 inch2, NKP M3 mouse cage 33 x 15 x 13cm, NKP RC2R rat cage 56 x 38x 17 (cages can be joined together), Thoren small cage filter top 19.56 x 30.91 x 13.34cm, Thoren large cage filter top 30.80 x 30.80 x 14.29cm, Allentown rat cage 588 mm x 286 mm x 412 mm

**Food and water (type, composition, supplier and access)**	Free feeding Irradiated 5LF2 = RM1 & Irr 5LFJ = RM3 both extruded diet supplier (IPS). Automatic watering system	Free feeding Irradiated 5LF2 = RM1 & Irr 5LFJ = RM3 both extruded diet supplier (IPS). Water source autoclaved tap water for IVC cages. Open top cages have tap water unsterilised
**Bedding and nesting material**	Corn cob nestpaks bedding<br>Nesting material - Nestpak outer, sizzle + rodent rolls (DBM)	Bedding type EC0 - pure aspen chip 2HK (DBM). Nesting material - Sizzle + rodent rolls (DBM)<br>Nude mice bedding - Alpha dry+ paper shavings + sizzle (DBM). Rats: paper wool, sizzle and hay (DBM)
**Temperature and humidity**	Temperature 19-21c Humidity 45-65%	Temperature 19-21c Humidity 45-65%
**Sanitation (frequency of cage/tank water changes, material transferred, water quality)**	All cages changed fortnightly. Spot changes in between.	IVC cages changed on a rota basis every other week. Open top and filter cages changed weekly. Water bottles changed weekly on all cage types.
**Social environment (group size and composition/stocking density)**	Depends on litter sizes and matings.<br>Single housed animals are avoided where possible.	Depends on study/cage type. Single housed animals are avoided.
**Biosecurity (level)**	Wet shower entry to facility. Disinfection chamber for all items, either Virkon or VHP for electrical items. Autoclave all cages, bedding, nesting materials, enrichment and garments.	Air shower in facility. Virkon to decontaminate smaller items. Large items decontaminated via vapourised hydrogen peroxide fogging.
**Lighting (type, schedule and intensity)**	12/12 7am to 7pm cycle	12/12 7am to 7pm cycle
**Environmental enrichment**	Rodent rolls, sizzle, carboard tunnels, cardboard houses, swings, sunflower seeds and wheat grains.	Standard cage Tecniplast blue line,Thoren IVC and Thoren filter top: Rodent rolls, sizzle, chew sticks, cardboard houses, red plastic tube, swings sunflower seeds or wheat grains.<br>Standard open top cage: Rodent rolls, sizzle, chew sticks, cardboard houses, red plastic tube, cable ties on roof of cage, sunflower seed or wheat<br>grains

		Rats standard cage: Paper wool, sizzle, hay, plastic red tubs on both the floor or cage and roof, chew sticks, sunflower seeds
**Sex of the experimenter/care staff**	Both male and female	Both male and female
**Weaning Age Range (Extra info above ARRIVE)**	21 days	19 to 24 days strain dependent
**Biopsy Age Range (Extra info above ARRIVE)**	21 to 28 days	21 to 28 days

13. Animal care and monitoring

a. Describe any interventions or steps taken in the experimental protocols to reduce pain, suffering and distress.
b. Report any expected or unexpected adverse events.
c. Describe the humane endpoints established for the study, the signs that were monitored and the frequency of monitoring. If the study did not have humane endpoints, state this.

All animals are checked daily by animal facility staff, this involves visually and physically observing the animals and their environment.

Need to expand this section to answer the questions….
Weighing, BCS
Any SC18 reports?
HEPs for the studies?
