# Supplementary material for: Endogenous LRRK2 and PINK1 function in a convergent neuroprotective ciliogenesis pathway in the brain
Source: Proc Natl Acad Sci U S A. 2025 Jan 28;122(5):e2412029122. doi: 10.1073/pnas.2412029122 (PMC11804522; doi:10.1073/pnas.2412029122)
Supplement: Supplementary file 2 — Appendix 02 (PDF) [file pnas.2412029122.sapp2.pdf]

SUPPLEMENTARY METHODS

Cell and tissue lysis

Cells were quickly washed on ice in PBS, then lysed in buffer containing Tris-HCl (50 mm, pH 7.5), EDTA (1 mM), EGTA (1 mM), Triton (1% w/v), sodium orthovanadate (1 mM), sodium glycerophosphate (10 mM), sodium fluoride (50 mM), sodium pyrophosphate (10 mM), sucrose (0.25 mM), protease inhibitor cocktail (Roche), phoSTOP (Roche), and chloroacetamide (200 mM). Tissues were instead collected and snap frozen in liquid nitrogen. They were then weighted, quickly thawed on ice in a 10-fold volume excess of ice cold lysis buffer. Tissues were homogenised using a POLYTRON homogenizer (KINEMATICA), employing three rounds of 10s homogenization with 10s intervals on ice. Lysates either from cells or tissues, were incubated for 30 min on ice. Samples were spun at 17000 g in an Eppendorf 5417R centrifuge for 30 min at 4°C. Supernatants were collected, and protein concentration was determined by using the Bradford kit (Pierce).

pSer65 Ub ELISA

Phosphorylation of Ub at Ser65 by PINK1 was monitored in vivo by enzyme linked immunosorbent assay (ELISA) as previously described by Watzlawik and colleagues [55] and detailed in dx.doi.org/10.17504/protocols.io.n2bvjnq3bgk5/v1. Briefly, MSD plates were coated overnight with 30 µl/well of 200 mM sodium carbonate buffer (pH 9.7) containing 1 µg/ml of rabbit monoclonal pSer65-Ub antibody. The next morning plates were washed twice with ELISA washing buffer (150 mM Tris, pH 7.4, 150 mM NaCl, 0.1% Tween-20) by plate inversion and gentle tapping on paper towels (not by pipette aspiration). Plates were then blocked with ELISA blocking buffer (150 mM Tris, pH 7.4, 150 mM NaCl,0.1% Tween-20, 1% BSA) for 1h at room temperature. All samples were run in duplicates and diluted in blocking buffer. 30 µg of total protein were loaded per well for all mouse tissues in a total volume of 30 µl per well. Detergent volumes were adjusted across all samples. Antigens were incubated for 2 h at room temperature on a microplate mixer at 600 rpm and three washing steps were then performed as described before. Mouse total Ub antibody (clone P4D1; Thermo Fisher #14-6078-37) was subsequently added as detecting antibody at a final concentration of 5 µg/ml in blocking buffer in 30 µl total volume per well. After three washing steps, 50 µl/well of 1 µg/ml of SULFO-TAG labelled goat anti-mouse antibody (MSD, R32AC-1) in blocking buffer were added and incubated for 1 h at room temperature on a microplate mixer at 500 rpm. After another three washing steps, 150 µl MSD GOLD Read Buffer (MSD, R92TG-2) were finally added to each well and the plate read on a MESO QuickPlex SQ 120 reader.

MEF generation, maintenance, and treatment

Mouse embryonic fibroblasts (MEFs) were isolated from mice embryos of different genotype and relative littermate WT as extensively described in dx.doi.org/10.17504/protocols.io.eq2ly713qlx9/v1 and then immortalised by SV40-mediated immortalization. MEFs were cultured in DMEM (Dulbecco's modified Eagle's medium) high glucose supplemented with 10% (v/v) FBS, 2 mM L-glutamine, Penicillin-Streptomycin 100U/mL, 1 mM Sodium Pyruvate, 1X non-essential amino acids solution. To induce mitochondrial depolarization, MEFs were treated for 24h with a combination of Oligomycin and Antimycin A at a final concentration of 1 µM and 10 µM. The type-2 LRRK2 inhibitor MLi-2 was used at a final concentration of 100 nM for 1h and 30 min. Inhibition of transcription and translation was achieved by using 5,6-dichlorobenzimidazole (DRB) at 100 µM and cycloheximide at 1 µg/ml for 24h, both dissolved and diluted in DMSO. For the panel of mitochondrial stressors, 1x106 PPM1HWT MEFs were seeded per 10 cm2 dish. The day after, MEFs were treated with the different compounds for 24h before being lysed. All compounds were dissolved and diluted in DMSO, except for sodium selenite and DFP, diluted in H2O. The details of the compounds and the concentrations used can be found in the supplementary and in the legend of figure 4.

SUPPLEMENTARY FIGURE LEGENDS

Supp Figure 1

New Rab12 antibody validation in Rab12$^{+/+}$, Rab12$^{+/-}$ and Rab12$^{-/-}$ mice and cell lines. ***A.*** Immunoblot of large intestine, brain and ***B.*** kidney, lung from Rab12 wild type, heterozygous and homozygous Knock-out, demonstrating the increase sensitivity of the new anti-Rab12 antibody compared to the previously available antibody. ***C.*** Western blot analysis of lysates from WT and Rab12 KO A549 and mouse embryonic fibroblast (MEF)
The ratio of Rab12/vinculin is plotted in the graphs for the large intestine, brain, kidney and lung in ***D., E., F., G.***, respectively. Each lane is loaded with 40 µg of protein lysate from the tissue or 15 µg from cell lysates. Ordinary one-way ANOVA with Dunnett's multiple comparison test versus Rab12$^{+/+}$. * $p<0.05$, ** $p<0.01$, *** $p<0.001$, **** $p<0.0001$. The new antibody, Rab12 Rabbit mAb MJFF-A-6E6 (A26172) was used at 0.05 ug/ml dilution.

Supp Figure 2

LRRK2 signalling in the olfactory bulb and hippocampus is not affected by loss of PINK1 *in vivo. A.* Immunoblot of LRRK2 pathway component in mouse olfactory bulb and

B. hippocampus. Quantification of ***C****.* pSer105/total Rab12, ***D****.* PPM1H/Vinculin and ***E.*** pSer935/total LRRK2 for the olfactory bulb and in ***F., G., H.*** for the hippocampus. Each lane was loaded with 40 µg of protein lysate from one mouse. In graphs, black circle represents PINK1^{WT} while red square PINK1^{KO} animals. Box and whiskers plot, from min to max with the median line. Ordinary 2-way ANOVA with Sidak's multiple comparison test. * p<0.05, ** p<0.01, *** p<0.001, **** p<0.0001.

Supp Figure 3

LRRK2 signalling in the midbrain and thalamus is not affected by loss of PINK1 *in vivo.* ***A.*** Immunoblot of LRRK2 pathway component in mouse midbrain and ***B.*** thalamus. Quantification of ***C****.* pSer105/total Rab12, ***D****.* PPM1H/Vinculin and ***E.*** pSer935/total LRRK2 for the midbrain and in ***F., G., H.*** for the thalamus. Each lane was loaded with 40 µg of protein lysate from one mouse. In graphs, black circle represents PINK1^{WT} while red square PINK1^{KO} animals. Box and whiskers plot, from min to max with the median line. Ordinary 2-way ANOVA with Sidak's multiple comparison test. * p<0.05, ** p<0.01, *** p<0.001, **** p<0.0001.

Supp Figure 4

LRRK2 signalling in the cerebellum and brainstem is not affected by loss of PINK1 *in vivo.* ***A.*** Immunoblot of LRRK2 pathway component in mouse cerebellum and ***B.*** brainstem. Quantification of ***C****.* pSer105/total Rab12, ***D****.* PPM1H/Vinculin and ***E.*** pSer935/total LRRK2 for the cerebellum and in ***F., G., H.*** for the brainstem. Each lane was loaded with 40 µg of protein lysate from one mouse. In graphs, black circle represents PINK1^{WT} while red square PINK1^{KO} animals. Box and whiskers plot, from min to max with the median line. Ordinary 2-way ANOVA with Sidak's multiple comparison test. * p<0.05, ** p<0.01, *** p<0.001, **** p<0.0001.

Supp Figure 5

LRRK2 signalling in the spinal cord is not affected by loss of PINK1 *in vivo.* ***A.*** Immunoblot of LRRK2 pathway component in mouse spinal cord. Quantification of ***B.*** pSer105/total Rab12, ***C.*** PPM1H/Vinculin and ***D.*** pSer935/total LRRK2. Each lane was loaded with 40 µg of protein lysate from one mouse. In graphs, black circle represents PINK1^{WT} while red square PINK1^{KO} animals. Box and whiskers plot, from min to max with the median line. Ordinary 2-way ANOVA with Sidak's multiple comparison test. * p<0.05, ** p<0.01, *** p<0.001, **** p<0.0001.

Supp Figure 6

LRRK2 signalling in the lung and spleen is not affected by loss of PINK1 *in vivo. A*. Immunoblot of LRRK2 pathway component in mouse lungs and relative quantification of ***B.*** pThr73/total Rab10, ***C.*** pSer105/total Rab12, ***D.*** pSer935/total LRRK2, and ***F.*** PPM1H/Vinculin. Similarly in ***F., G., H.***, and ***I.*** analysis from mouse spleen. pSer105 Rab12 was undetectable in this tissue. Each lane was loaded with 40 μg of proteins lysate from one mouse. In graphs, black circle represents $PINK1^{WT}$ while red square $PINK1^{KO}$ animals. Box and whiskers plot, from min to max with the median line. Ordinary 2-way ANOVA with Sidak's multiple comparison test. * p<0.05, ** p<0.01, *** p<0.001, **** p<0.0001.

Supp Figure 7

Behavioural testing sub-analysis in 10.5 months old double mutant PINK1 knockout / LRRK2 R1441C mutant mice does not suggest genetic interaction *in vivo*. *A.* Forelimb and *B.* hindlimb paws base width during gait analysis. The overlap of the two is shown in graph C. The average amount of footslips after two balance beam tests is shown in D. for forelimbs. E. for hindlimb and their sum in F. Weight at 10.5 months separated by sex of the animal for females G. and males H. In violin plots, black circle represents LRRK2WT while red squares LRRK2RC mice. Ordinary 2-way ANOVA with Sidak's multiple comparison test. * p<0.05, ** p<0.01, *** p<0.001, **** p<0.0001. N=15/16 mice per group

Supp Figure 8

Immunohistochemistry analysis of medium spiny neurons in double mutant PINK1 knockout / LRRK2 R1441C mutant mice. ***A.*** Representative images and ***B.*** quantification of DARPP32 staining and ***C.*** striatal volume in the brain of 10.5 months old mice. In bar charts, black circle represents $LRRK2^{WT}$ while red squares $LRRK2^{RC}$ mice. Ordinary 2-way ANOVA with Sidak's multiple comparison test. N=15/16 mice per group. Scale bar 50 μm.

Supp Figure 9

pSer65 Ubiquitin analysis in double mutant PINK1 knockout / LRRK2 R1441C mutant mice. Detection of pSer65 Ub by ELISA in different regions of the CNS such as cortex ***A.***, midbrain ***B.***, cerebellum ***C.*** and spinal cord ***D.*** In box and whiskers plots, black circle represents $LRRK2^{WT}$ while red square $LRRK2^{RC}$ mice. Ordinary 2-way ANOVA with Tukey's multiple comparison test. N=4 mice per group.

Supp Figure 10

Mitochondrial depolarisation induce PPM1H increase in primary MEFs. ***A.*** Immunoblot of two independent MEF clones upon 24h treatment with O/A, 2h treatment with MLi-2. ***B.*** Quantification of PPM1H, ***C.*** pThr73 Rab10 and ***E.*** pSer935 LRRK2. Each lane was loaded with 20 µg of protein lysates and the experiment performed twice with a total of N=4 independent clones. Graphs show mean ± SEM, ordinary 1-way ANOVA with Dunnett's multiple comparison test vs DMSO group. * p<0.05, ** p<0.01, *** p<0.001, **** p<0.0001.

Supp Figure 11

Analysis of LRRK2 and PINK1 Rab phosphorylation in primary MEFs derived from double mutant PINK1 knockout / LRRK2 R1441C mutant mice. *A.* Immunoblot of two independent MEF clones from WT/WT, KO/WT, WT/RC and KO/RC mice upon 24h treatment with O/A, 2h treatment with MLi-2. *B.* Quantification of pSer111 and *C.* pThr72 Rab8A following Rab8A immunoprecipitation. *D.* Quantification of pThr73 Rab10 and *E.* pSer935 LRRK2. In graphs, black circles represent $LRRK2^{WT}$ while red square $LRRK2^{RC}$ samples. Graphs show mean ± SEM from N=2 clones per genotype.

Supp Figure 12

Mitochondrial depolarisation-induced PPM1H increase is independent of PINK1 and LRRK2 in MEFs. Immunoblot from ***A.*** $PPM1H^{WT}$ and $PPM1H^{KO}$, ***B.*** $LRRK2^{WT}$ and $LRRK2^{KO}$ and ***C.*** $PINK1^{WT}$ and $PINK1^{KO}$ MEFs upon 24h treatment with O/A, 2h treatment with MLi-2 or the combination of both. Quantification of ***D., E., F.,*** PPM1H ***G., H., I.,*** pThr73 Rab10 and ***J., K., L.*** pSer935 LRRK2. Each lane was loaded with 40 µg of protein lysates. Graphs show mean ± SEM from N=1 experimental replicate with two biological replicates.

Supp Figure 13

PPM1H protein and mRNA dynamics upon mitochondrial damage. Quantification of ***A.*** pSer935/tot LRRK2 and ***B.*** pThr73/tot Rab10 protein levels in $PPM1H^{WT}$ MEFs upon 0h, 4h, 8h, 16h, and 24h treatment with O/A. ***C.*** Dynamics of PPM1H mRNA (other pair of primers) and of ***D.*** ATF4 during increasing times of mitochondria depolarization. ***E.*** Schematic of experimental methodology followed to depolarize mitochondria and inhibit transcription (DRB) or translation (CHX) in $PPM1H^{WT}$ and $PPM1H^{KO}$ MEFs. ***G.*** PPM1H mRNA levels upon treatment with O/A and DRB or CHX using a different pair of primer. Ordinary 2-way

ANOVA with Tukey's multiple comparison test from three independent experiments. Empty circles denote DMSO control while black circles represent O/A treated samples.

SUPPLEMENTARY FIGURES

Supp1

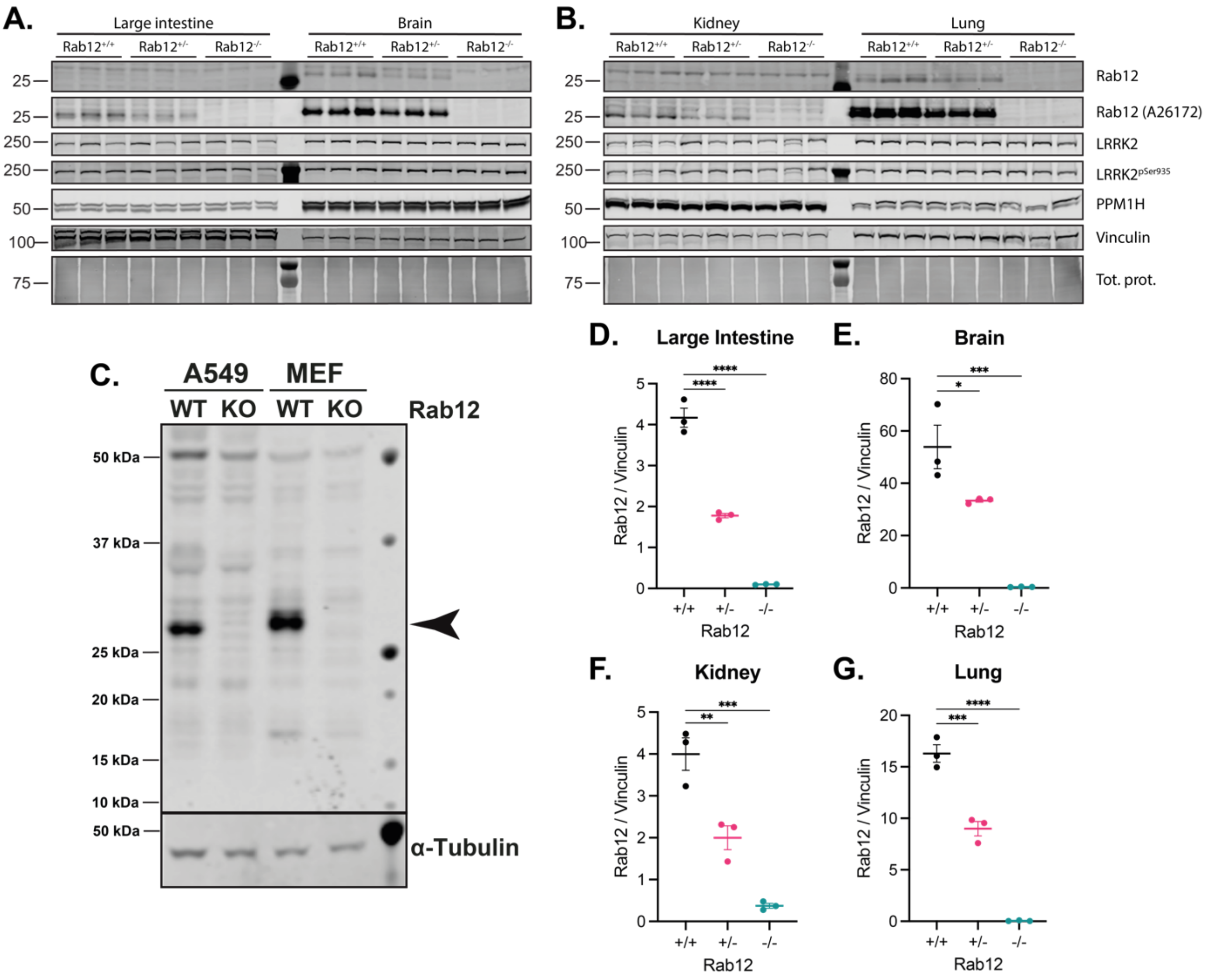

Supp2

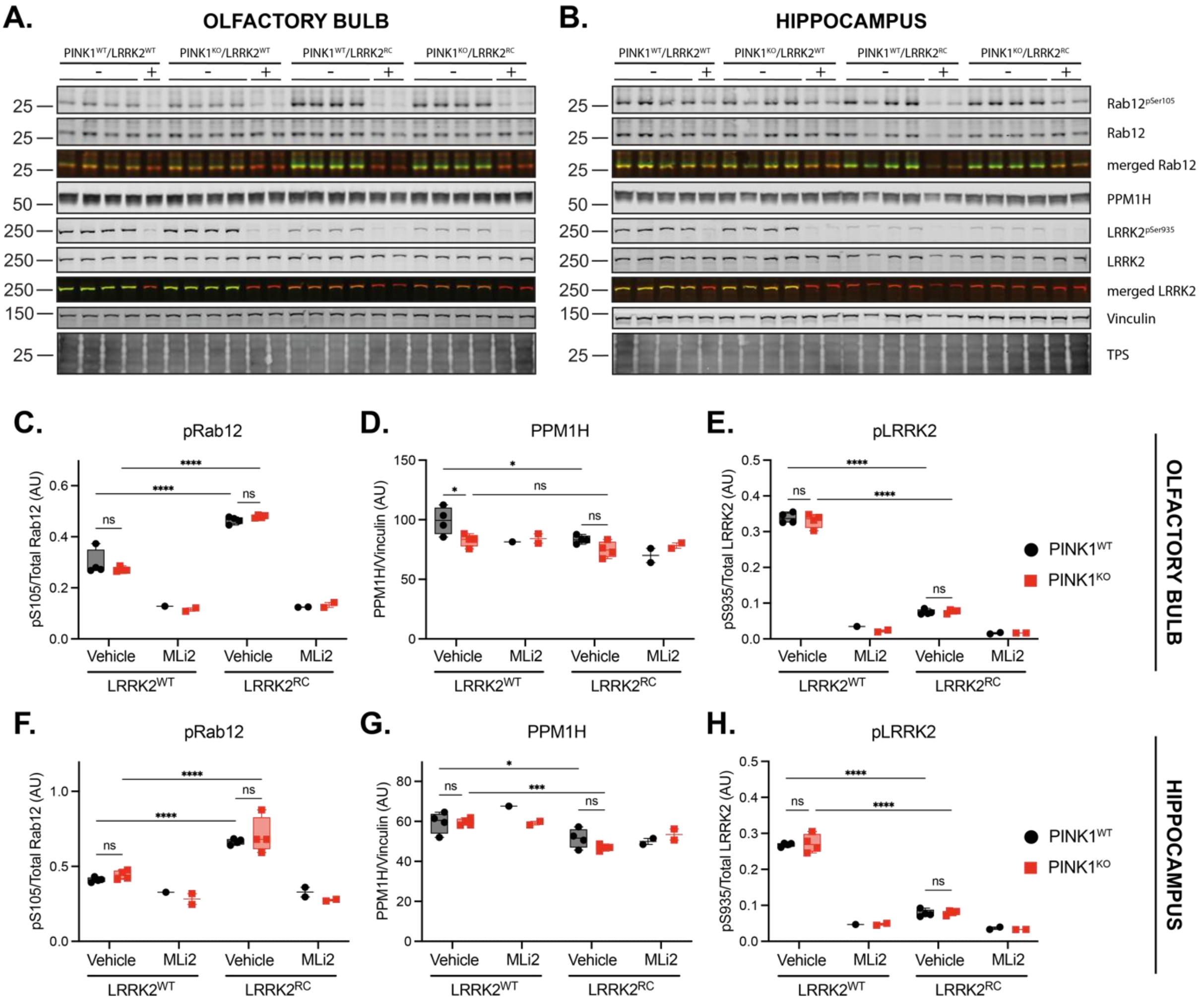

A.
OLFACTORY BULB
B.
HIPPOCAMPUS
PINK1WT/LRRK2WT
PINK1KO/LRRK2WT
PINK1WT/LRRK2RC
PINK1KO/LRRK2RC
Rab12pSer105
Rab12
merged Rab12
PPM1H
LRRK2pSer935
LRRK2
merged LRRK2
Vinculin
TPS
C.
pRab12
pS105/Total Rab12 (AU)
D.
PPM1H
PPM1H/Vinculin (AU)
E.
pLRRK2
pS935/Total LRRK2 (AU)
Vehicle
MLi2
LRRK2WT
LRRK2RC
PINK1WT
PINK1KO
OLFACTORY BULB
F.
pRab12
G.
PPM1H
H.
pLRRK2
HIPPOCAMPUS

Supp3

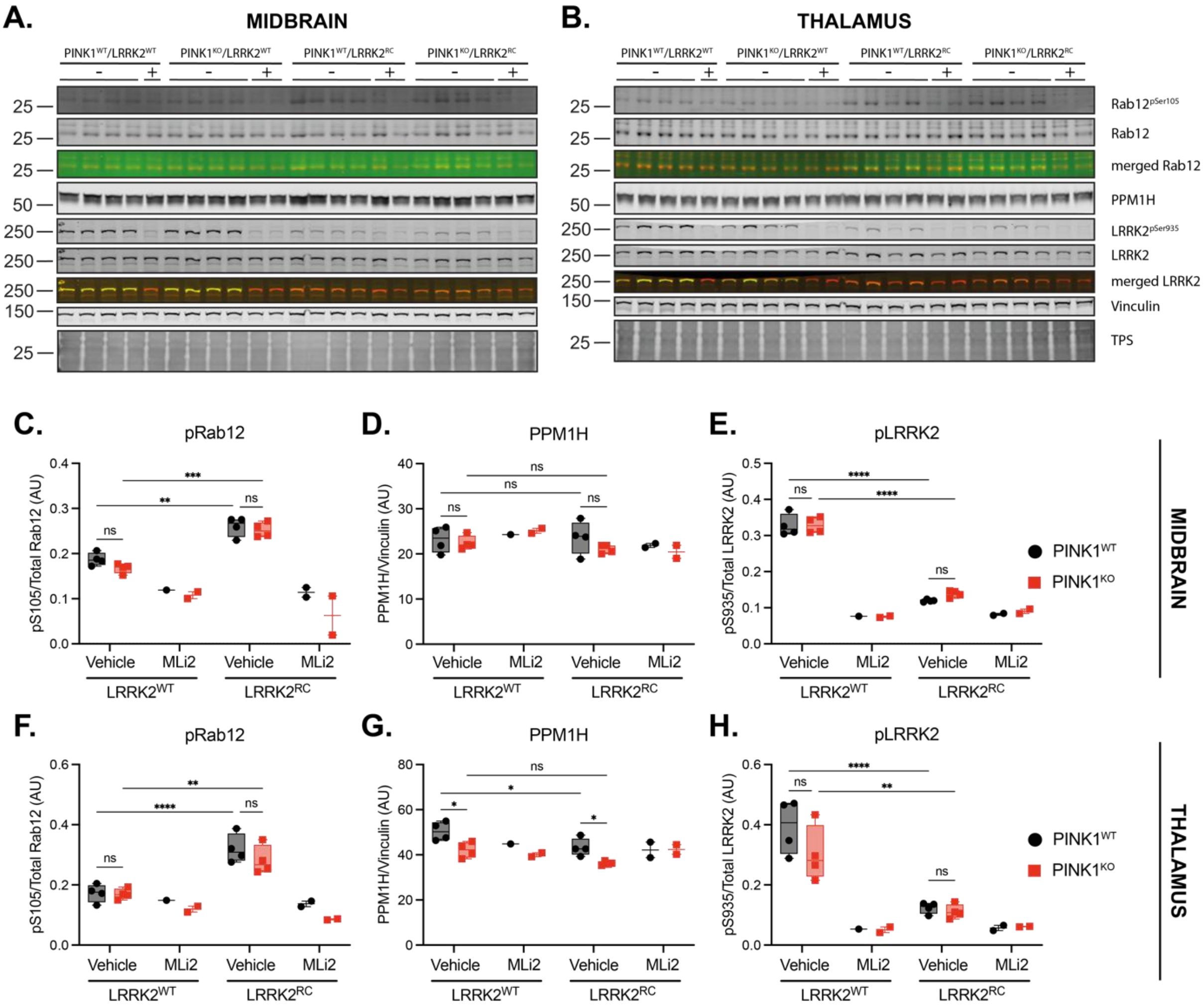

A.
MIDBRAIN
B.
THALAMUS
PINK1WT/LRRK2WT
PINK1KO/LRRK2WT
PINK1WT/LRRK2RC
PINK1KO/LRRK2RC
Rab12pSer105
Rab12
merged Rab12
PPM1H
LRRK2pSer935
LRRK2
merged LRRK2
Vinculin
TPS
C.
pRab12
D.
PPM1H
E.
pLRRK2
F.
pRab12
G.
PPM1H
H.
pLRRK2
pS105/Total Rab12 (AU)
PPM1H/Vinculin (AU)
pS935/Total LRRK2 (AU)
Vehicle
MLi2
LRRK2WT
LRRK2RC
PINK1WT
PINK1KO
MIDBRAIN
THALAMUS

Supp4

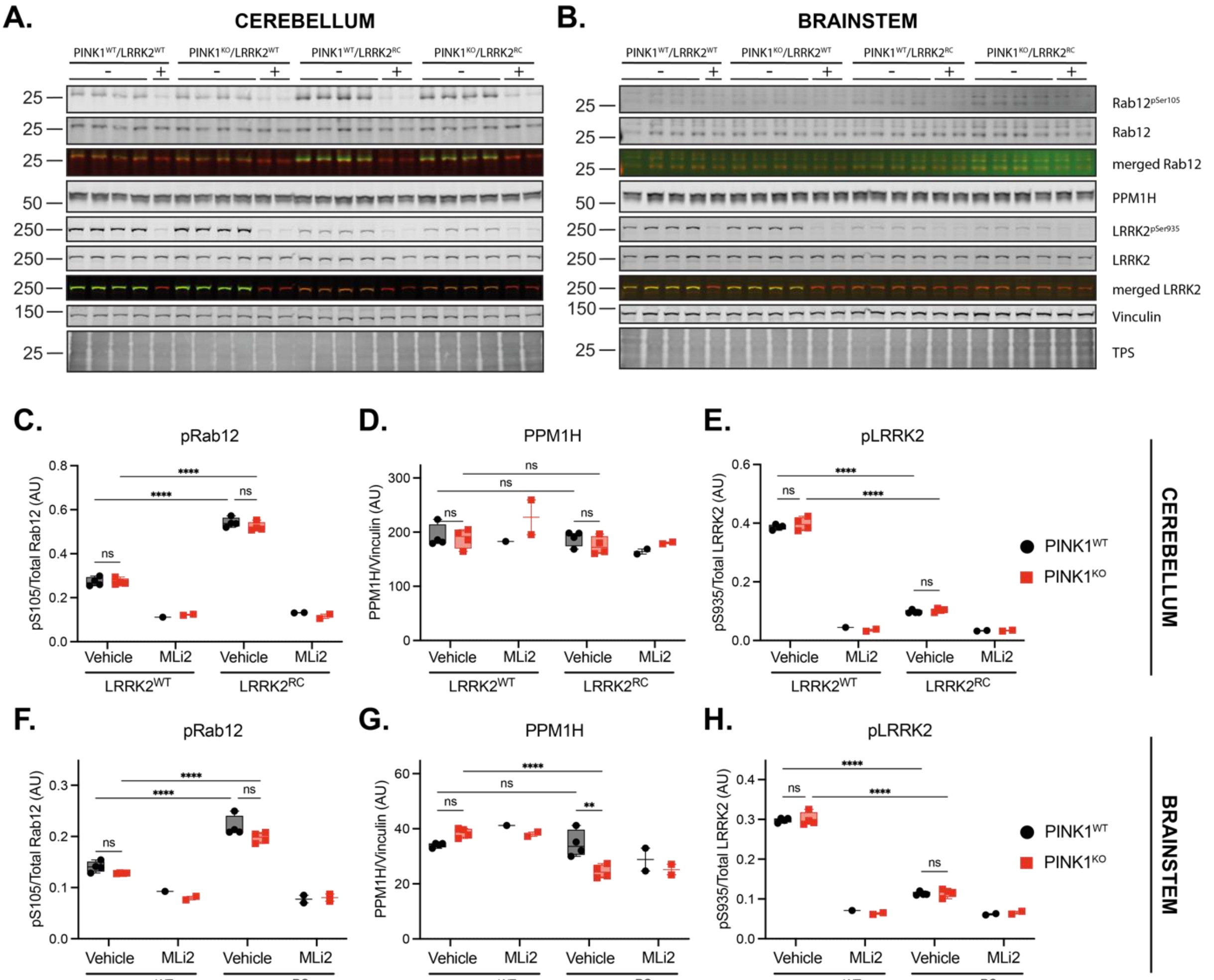

A.
CEREBELLUM
B.
BRAINSTEM
PINK1WT/LRRK2WT
PINK1KO/LRRK2WT
PINK1WT/LRRK2RC
PINK1KO/LRRK2RC
Rab12pSer105
Rab12
merged Rab12
PPM1H
LRRK2pSer935
LRRK2
merged LRRK2
Vinculin
TPS
C.
pRab12
pS105/Total Rab12 (AU)
D.
PPM1H
PPM1H/Vinculin (AU)
E.
pLRRK2
pS935/Total LRRK2 (AU)
F.
pRab12
G.
PPM1H
H.
pLRRK2
Vehicle
MLi2
LRRK2WT
LRRK2RC
PINK1WT
PINK1KO
CEREBELLUM
BRAINSTEM

Supp5

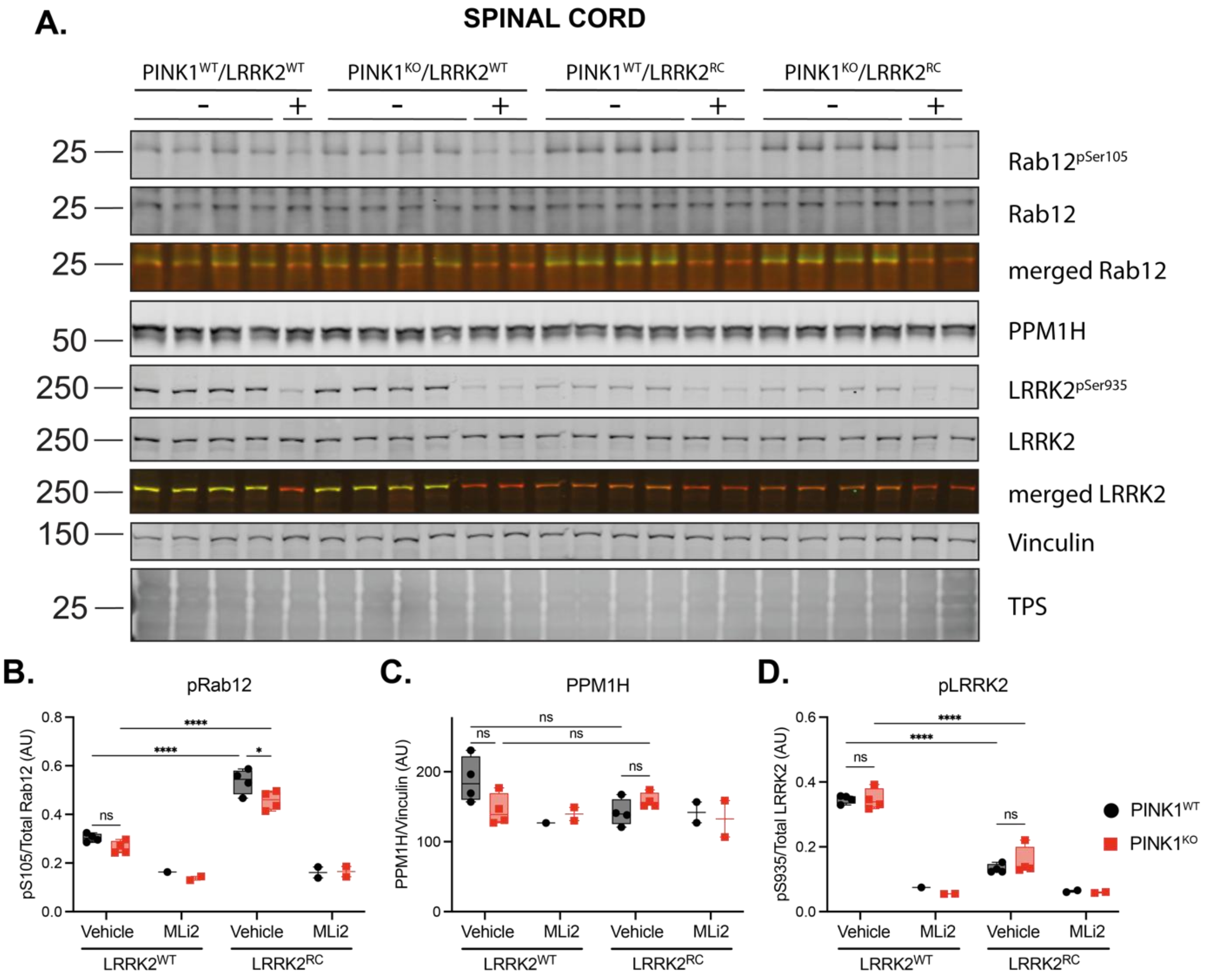

A.
SPINAL CORD
PINK1WT/LRRK2WT
PINK1KO/LRRK2WT
PINK1WT/LRRK2RC
PINK1KO/LRRK2RC
-
+
25
50
250
150
Rab12pSer105
Rab12
merged Rab12
PPM1H
LRRK2pSer935
LRRK2
merged LRRK2
Vinculin
TPS
B.
pRab12
pS105/Total Rab12 (AU)
C.
PPM1H
PPM1H/Vinculin (AU)
D.
pLRRK2
pS935/Total LRRK2 (AU)
ns
****
*
Vehicle
MLi2
LRRK2WT
LRRK2RC
PINK1WT
PINK1KO

Supp6

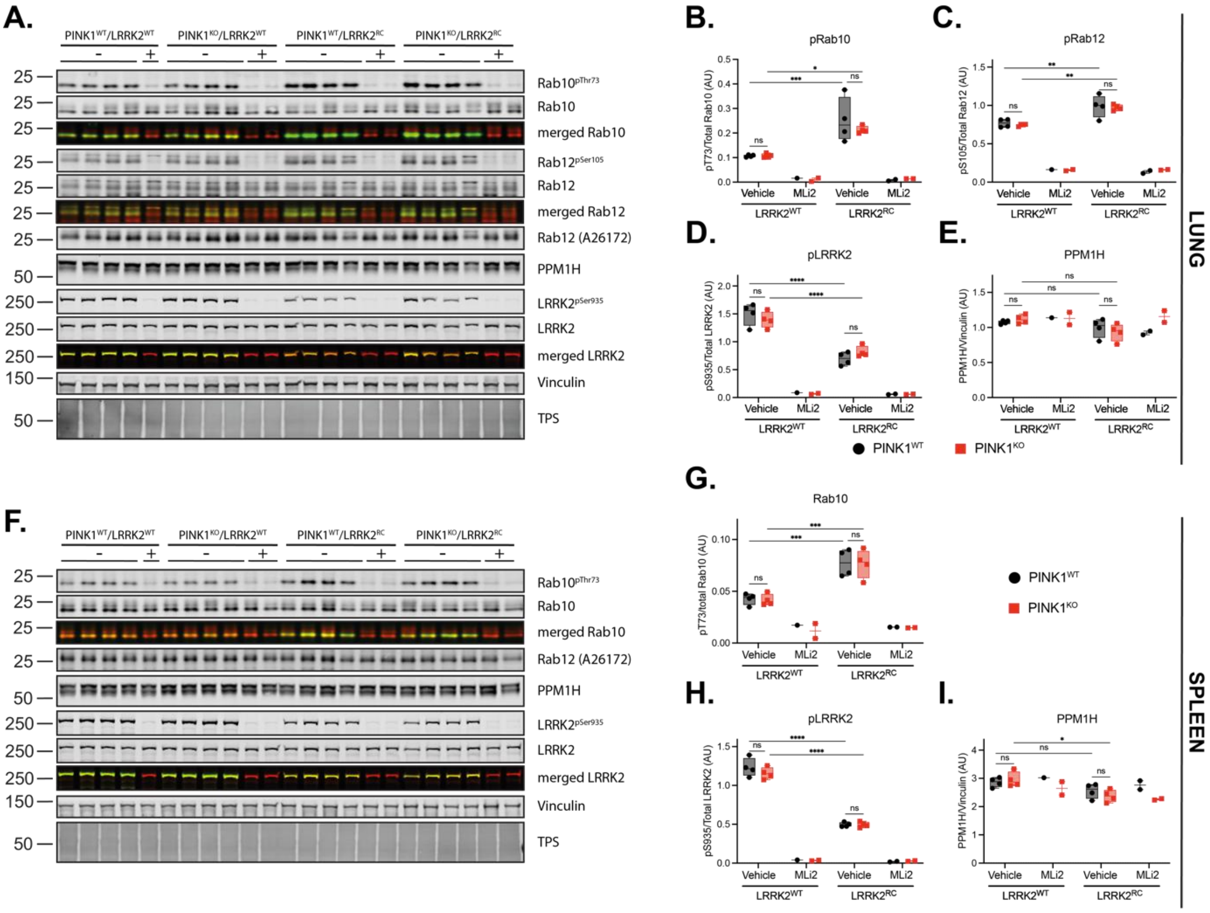
A.
B.
C.
D.
E.
F.
G.
H.
I.
pRab10
pRab12
pLRRK2
PPM1H
Rab10
LUNG
SPLEEN
PINK1WT
PINK1KO
Rab10pThr73
Rab10
merged Rab10
Rab12pSer105
Rab12
merged Rab12
Rab12 (A26172)
PPM1H
LRRK2pSer935
LRRK2
merged LRRK2
Vinculin
TPS
Vehicle
MLi2
LRRK2WT
LRRK2RC

Supp7

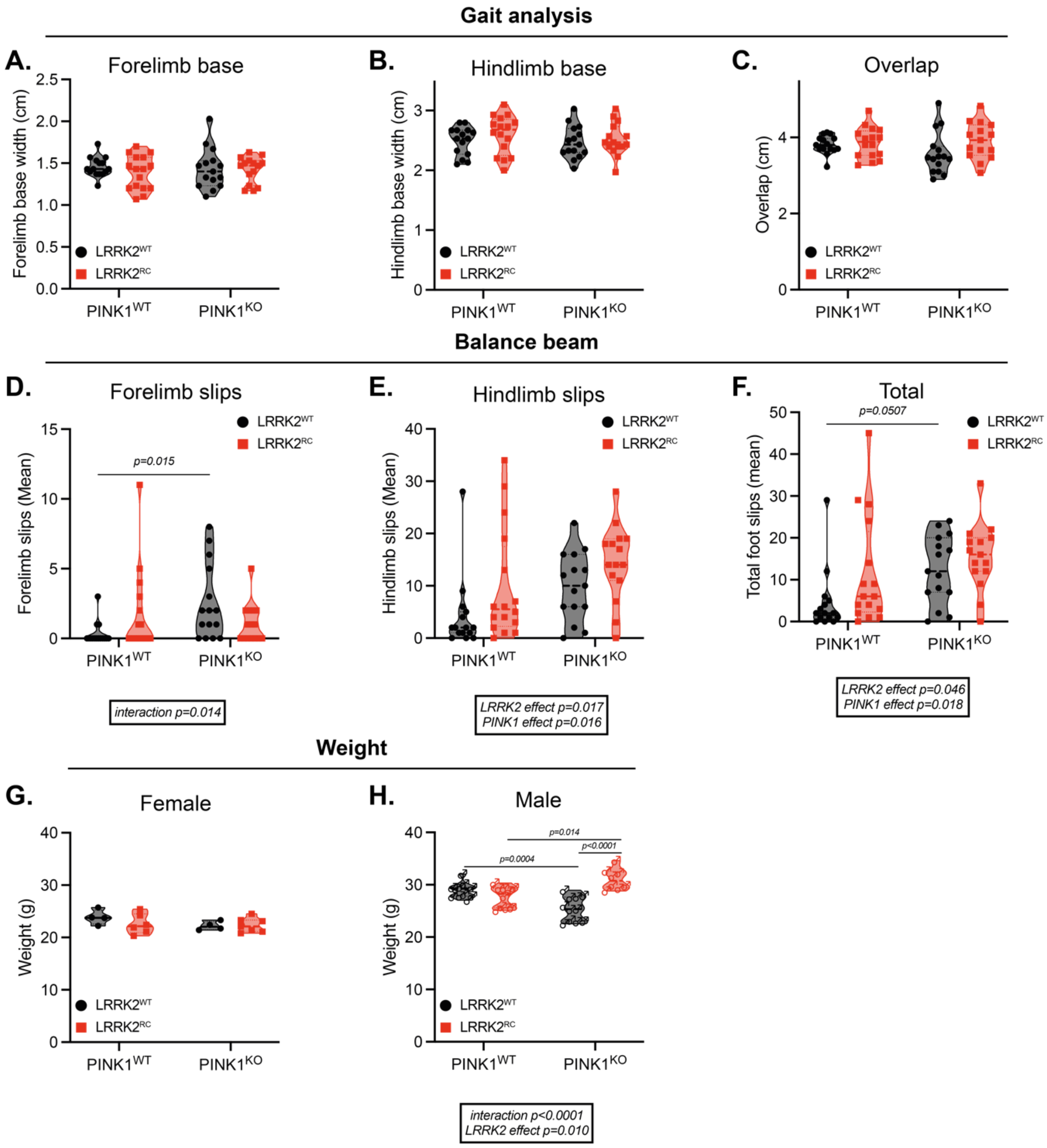

Gait analysis
A.
Forelimb base
Forelimb base width (cm)
LRRK2WT
LRRK2RC
PINK1WT
PINK1KO
B.
Hindlimb base
Hindlimb base width (cm)
C.
Overlap
Overlap (cm)
Balance beam
D.
Forelimb slips
Forelimb slips (Mean)
p=0.015
interaction p=0.014
E.
Hindlimb slips
Hindlimb slips (Mean)
LRRK2 effect p=0.017
PINK1 effect p=0.016
F.
Total
Total foot slips (mean)
p=0.0507
LRRK2 effect p=0.046
PINK1 effect p=0.018
Weight
G.
Female
Weight (g)
H.
Male
p=0.014
p<0.0001
p=0.0004
interaction p<0.0001
LRRK2 effect p=0.010

Supp8

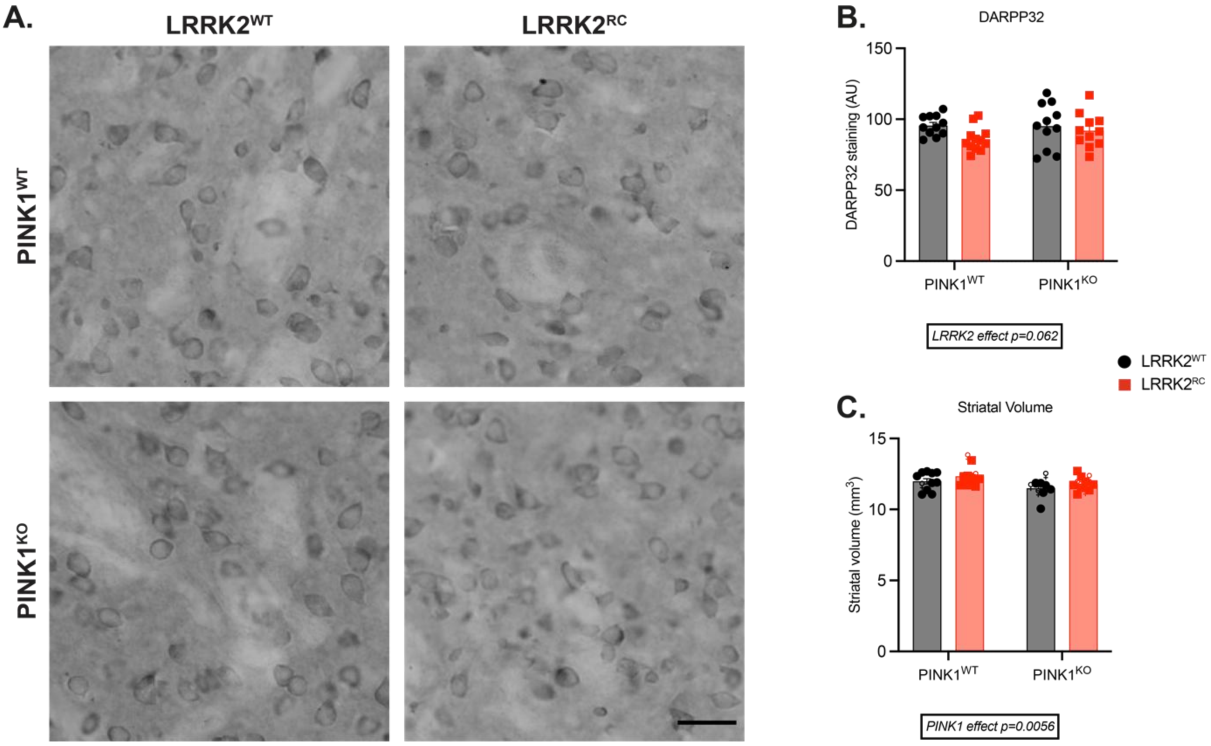

A.
LRRK2WT
LRRK2RC
PINK1WT
PINK1KO
B.
DARPP32
DARPP32 staining (AU)
150
100
50
0
PINK1WT
PINK1KO
LRRK2 effect p=0.062
LRRK2WT
LRRK2RC
C.
Striatal Volume
Striatal volume (mm3)
15
10
5
0
PINK1WT
PINK1KO
PINK1 effect p=0.0056

Supp9

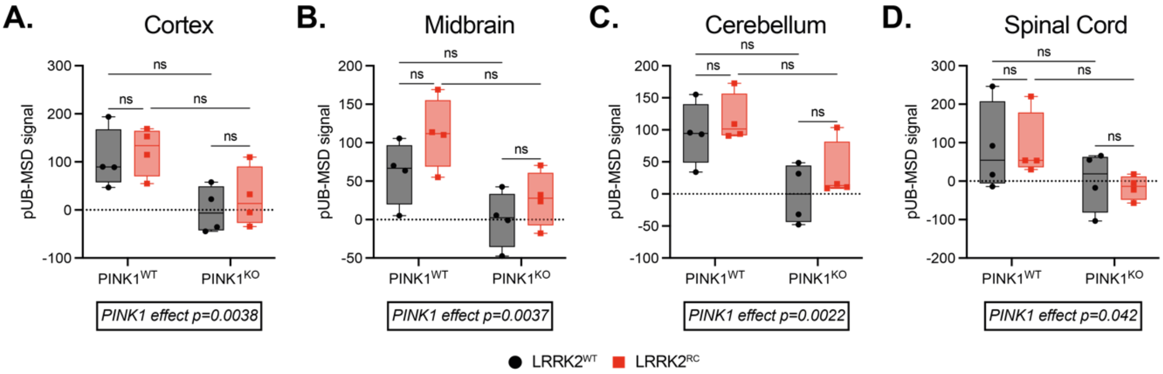

A.
Cortex
B.
Midbrain
C.
Cerebellum
D.
Spinal Cord
pUB-MSD signal
ns
PINK1WT
PINK1KO
PINK1 effect p=0.0038
PINK1 effect p=0.0037
PINK1 effect p=0.0022
PINK1 effect p=0.042
LRRK2WT
LRRK2RC

Supp10

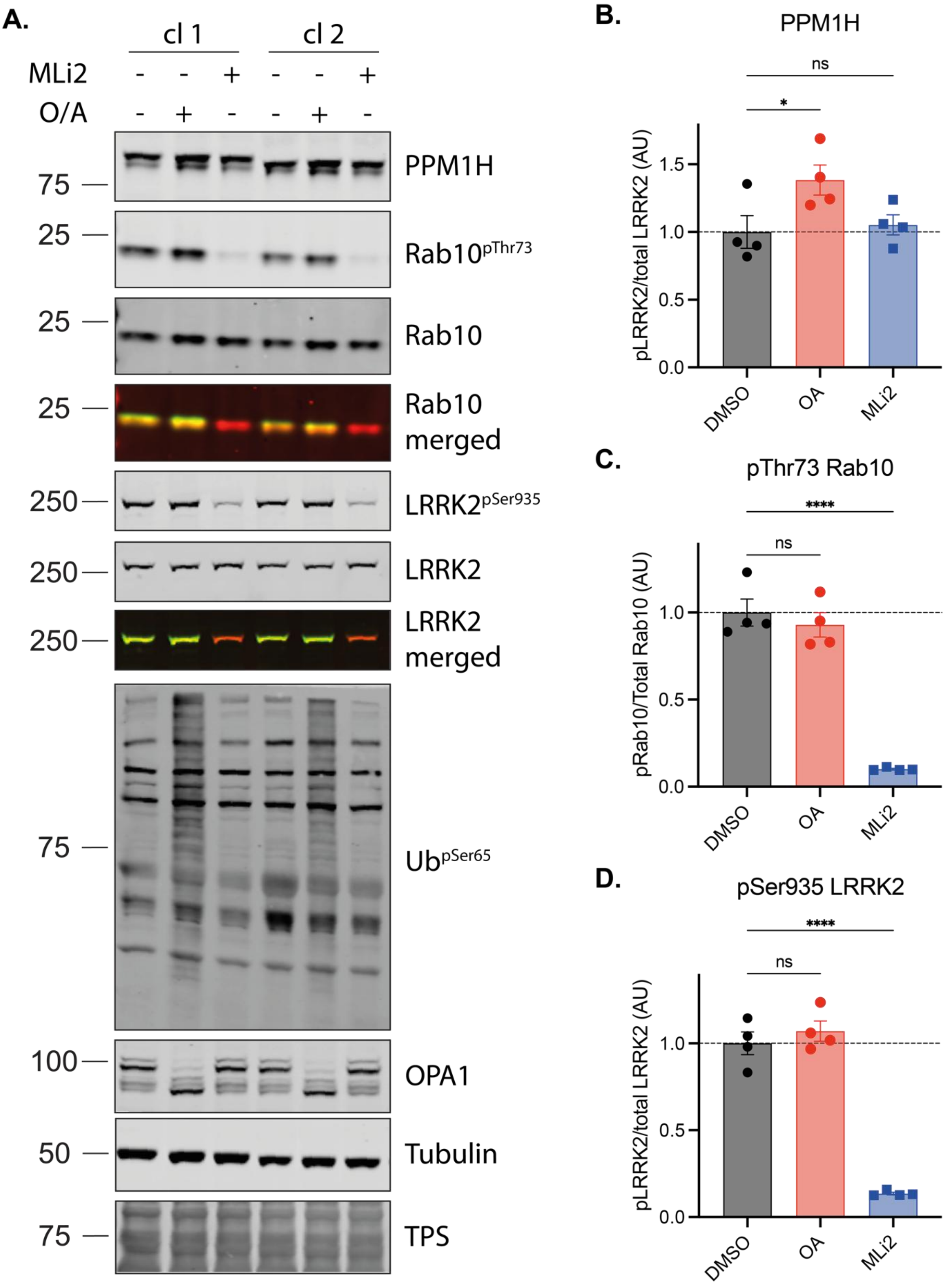
A.
cl 1
cl 2
MLi2 - - + - - +
O/A - + - - + -
75
25
25
25
250
250
250
75
100
50
75
PPM1H
Rab10pThr73
Rab10
Rab10 merged
LRRK2pSer935
LRRK2
LRRK2 merged
UbpSer65
OPA1
Tubulin
TPS
B.
PPM1H
ns
*
pLRRK2/total LRRK2 (AU)
0.0
0.5
1.0
1.5
DMSO
OA
MLi2
C.
pThr73 Rab10
****
ns
pRab10/Total Rab10 (AU)
0.0
0.5
1.0
DMSO
OA
MLi2
D.
pSer935 LRRK2
****
ns
pLRRK2/total LRRK2 (AU)
0.0
0.5
1.0
DMSO
OA
MLi2

Supp11

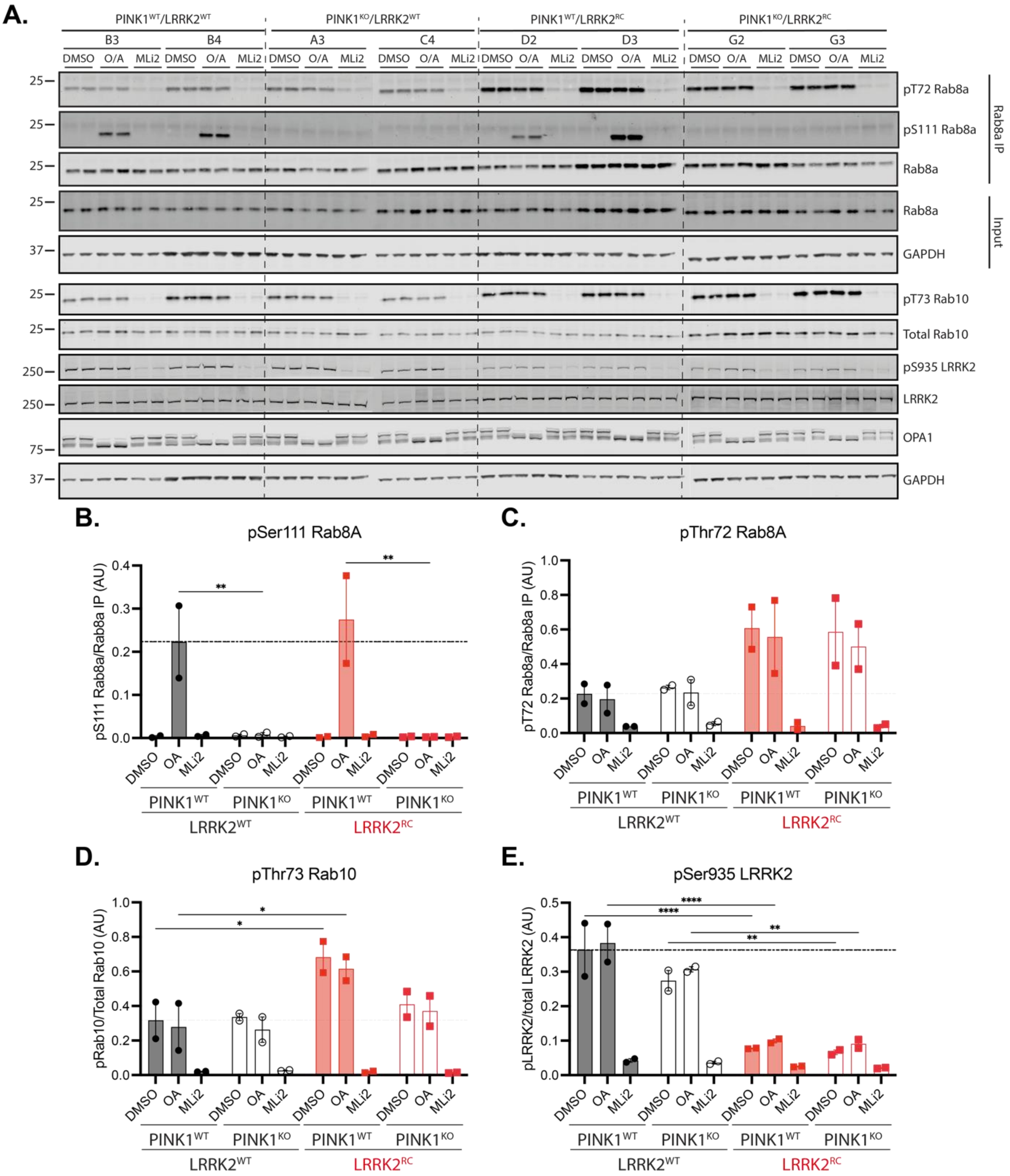
A.
PINK1WT/LRRK2WT
PINK1KO/LRRK2WT
PINK1WT/LRRK2RC
PINK1KO/LRRK2RC
B3 B4 A3 C4 D2 D3 G2 G3
DMSO O/A MLi2
pT72 Rab8a
pS111 Rab8a
Rab8a
Rab8a IP
Input
GAPDH
pT73 Rab10
Total Rab10
pS935 LRRK2
LRRK2
OPA1
25
37
250
75
B.
pSer111 Rab8A
pS111 Rab8a/Rab8a IP (AU)
C.
pThr72 Rab8A
pT72 Rab8a/Rab8a IP (AU)
D.
pThr73 Rab10
pRab10/Total Rab10 (AU)
E.
pSer935 LRRK2
pLRRK2/total LRRK2 (AU)
DMSO OA MLi2
PINK1WT PINK1KO
LRRK2WT
LRRK2RC
*
**
****

Supp12

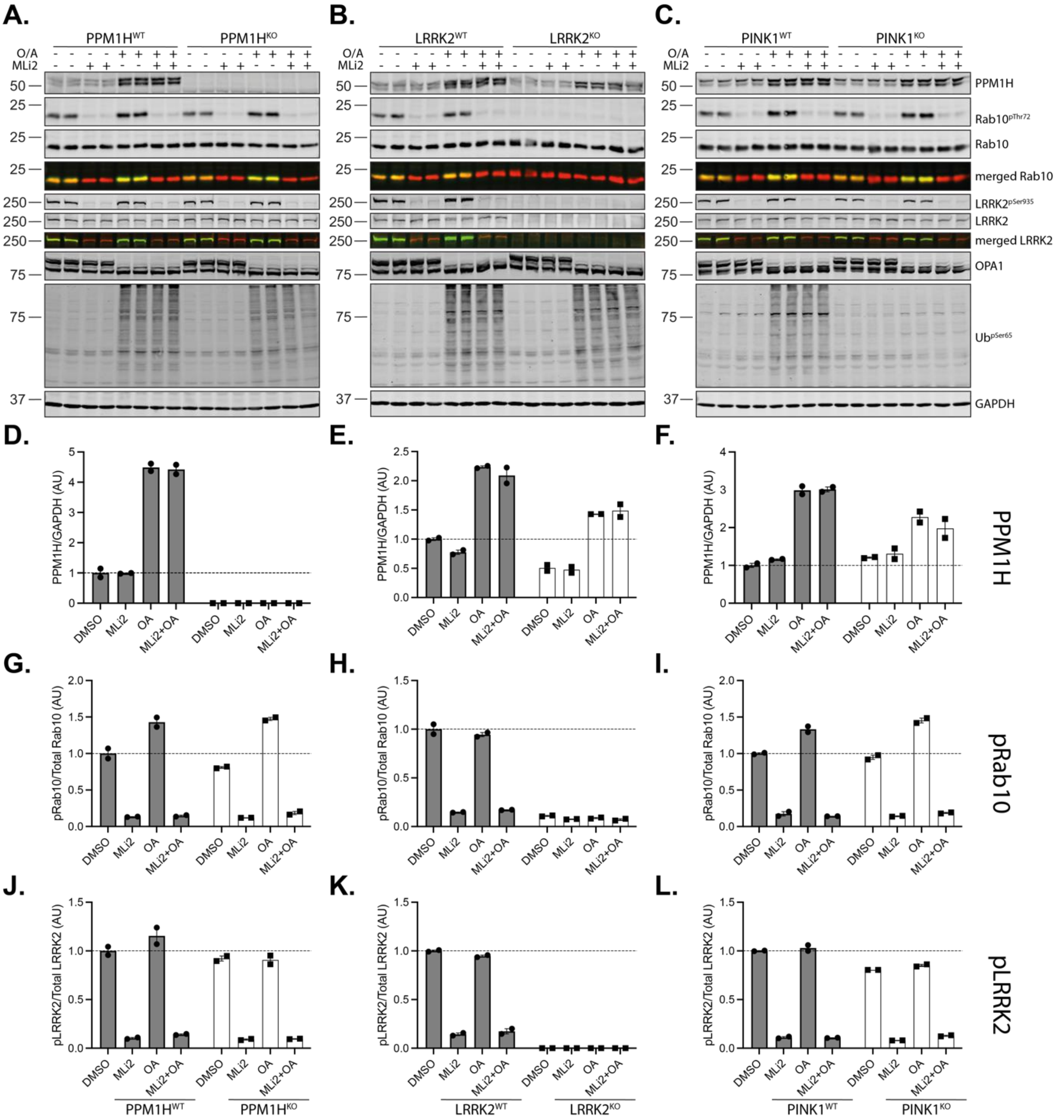

A.
PPM1H^{WT}
PPM1H^{KO}
B.
LRRK2^{WT}
LRRK2^{KO}
C.
PINK1^{WT}
PINK1^{KO}
O/A
MLi2
PPM1H
Rab10^{pThr72}
Rab10
merged Rab10
LRRK2^{pSer935}
LRRK2
merged LRRK2
OPA1
Ub^{pSer65}
GAPDH
D.
E.
F.
PPM1H/GAPDH (AU)
PPM1H
G.
H.
I.
pRab10/Total Rab10 (AU)
pRab10
J.
K.
L.
pLRRK2/Total LRRK2 (AU)
pLRRK2
DMSO
MLi2
OA
MLi2+OA

Supp13

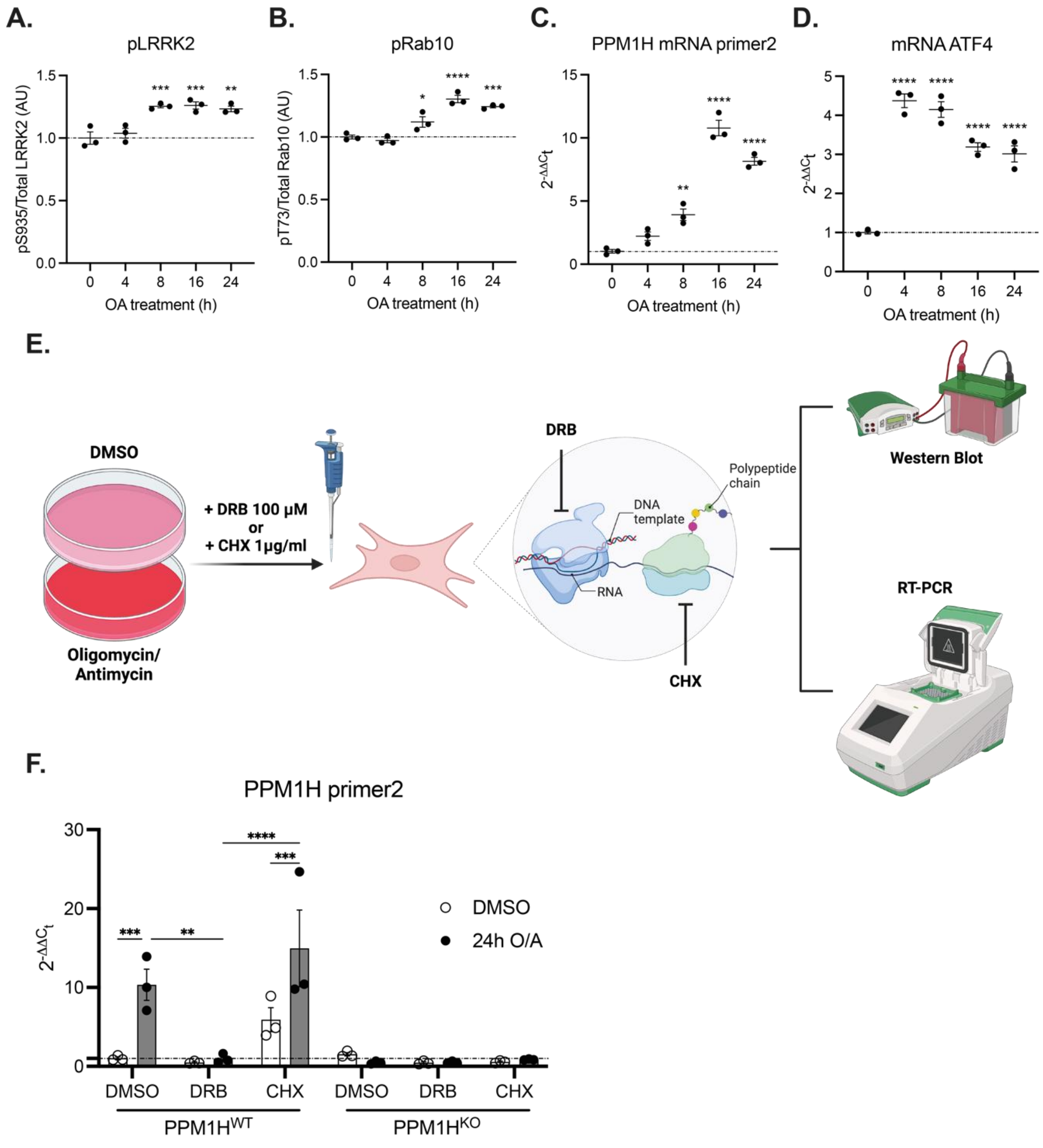

A.
pLRRK2
pS935/Total LRRK2 (AU)
1.5
1.0
0.5
0.0
0
4
8
16
24
OA treatment (h)
B.
pRab10
pT73/Total Rab10 (AU)
OA treatment (h)
C.
PPM1H mRNA primer2
OA treatment (h)
D.
mRNA ATF4
OA treatment (h)
E.
DMSO
Oligomycin/
Antimycin
+ DRB 100 µM
or
+ CHX 1µg/ml
DRB
DNA template
Polypeptide chain
RNA
CHX
Western Blot
RT-PCR
F.
PPM1H primer2
DMSO
24h O/A
DMSO
DRB
CHX
PPM1HWT
PPM1HKO
